# Outcomes of a tribal community program on hepatitis C, HIV, and syphilis screening, confirmation testing, and treatment initiation for an underserved population

**DOI:** 10.3389/fpubh.2025.1690448

**Published:** 2025-11-26

**Authors:** Ashley Comiford, Jorge Mera, Kendra Lewis, Savana Christy, Molly Feder, Andrea Blair

**Affiliations:** 1 Department of Infectious Diseases, Cherokee Nation Outpatient Health Center, Cherokee Nation Health Services, Tahlequah, OK, United States; 2 Sea Glass Group, Seattle, WA, United States

**Keywords:** American Indian, Alaska Native, hepatitis C, screening, community setting

## Abstract

**Background:**

American Indian and Alaska Native people are disproportionately impacted by hepatitis C, HIV, and syphilis, with rates 2.3, 1.9, and 6.4 times that of non-Hispanic White people, respectively. The objective was to describe the first 9 months implementing a community-based tribal program to screen and link underserved people to hepatitis C, HIV, and syphilis care and treatment. An additional objective was to identify patient characteristics associated with completing confirmation testing when indicated.

**Methods:**

This study occurred from January through September 2024 on the Cherokee Nation reservation in Oklahoma. Survey and medical record data from Cherokee Nation’s Hepatitis C Engagement and Linkage Program, a collaboration between the Cherokee Nation Health Services and community programs servicing people with substance use disorders and/or reduced healthcare access. People 18 years and older who visited the organizations during the project period and provided consent were eligible. Project outcomes, including point of care screening results, receipt of confirmation testing, test results, and treatment initiation, were assessed using counts and proportions. Associations between patient characteristics and completion of confirmation testing were assessed using Chi-square or Fisher’s exact tests.

**Results:**

The program screened 503 people, including 365 American Indian and Alaska Native people, for hepatitis C, HIV, and syphilis. Forty-five percent of participants reported lifetime injection drug use. Overall, 102 (20%) participants had a reactive hepatitis C antibody test, 36 (35%) of whom received confirmatory hepatitis C RNA testing. Sixteen people were diagnosed with hepatitis C infection, 12 (75%) of whom initiated treatment. Sixty participants (12%) had a reactive *Treponema pallidum* syphilis antibody test, 18 (30%) of whom received confirmatory testing. Seventeen people were diagnosed with untreated syphilis, 16 (94%) of whom initiated treatment. Less than 1% of participants had a reactive HIV antibody test, although specific numbers are suppressed due to a small sample size. Most people who were linked to care beyond antibody testing were American Indian and Alaska Native.

**Conclusion:**

Cherokee Nation successfully screened underserved individuals for hepatitis C, HIV, and syphilis with moderate success in further evaluation and treatment. Future interventions should include onsite treatment options to prevent barriers to accessing healthcare.

## 1 Introduction

Hepatitis C virus (HCV) infection is one of the leading causes of end-stage liver disease, hepatocellular carcinoma, and liver-related deaths globally (1, 2). In 2023, there were about 101,525 newly reported chronic HCV cases and 69,000 estimated acute HCV infections in the United States (U.S.) (3). American Indian and Alaska Native (AI/AN) people are disproportionately impacted by HCV, experiencing the highest rates of acute HCV compared with all other reported racial and ethnic groups, at 3.5 cases per 100,000 people in 2023 (3). In addition, AI/AN people experience disparities in other transmissible diseases, including human immunodeficiency virus (HIV) and syphilis. In 2023, HIV incidence among AI/AN people was almost twice that of non-Hispanic White people, at 9.8 versus 5.2 per 100,000 people. In 2023, the rates of primary and secondary syphilis and congenital syphilis were also higher among AI/AN people compared with non-Hispanic White people, at 58.2 versus 9.1 per 100,000 people and 680.8 versus 57.3 per 100,000 people, respectively (4, 5).

Despite advances in diagnostic tests and an effective cure, few insured persons in the U. S. with HCV infection receive timely Direct Acting Antiviral (DAA) treatment, and disparities in treatment exist (6). A major risk factor for acquiring HCV is substance use disorders (SUD), particularly among people who inject drugs (7, 8). Further, stigma related to SUD and injecting drugs may hinder individuals from seeking care at traditional clinics, making them an often underserved population in relation to access to healthcare (9–11). In 2023, a higher percentage of AI/AN people reported using any illicit drugs, methamphetamine, misusing prescription pain relievers and opioids, having an SUD, and needing SUD treatment in the past year than any other racial or ethnic group. These disparities may be associated with experiences of historical trauma which disproportionately impacts AI/AN communities (12–14). It is unsurprising that AI/AN individuals experience disparities in HCV, HIV, and syphilis as these transmissible diseases share risk factors for infection (15, 16).

Cherokee Nation Health Services (CNHS) implemented an HCV elimination program in 2015 to address HCV disparities among AI/AN people (17). As part of the HCV elimination program, CNHS developed several strategies to reduce HCV infections, including a universal screening policy, training healthcare personnel on screening practices, laboratory-triggered screening, and electronic health record reminders for healthcare personnel to order screening tests (17). Further, CNHS expanded clinical capacity for evaluation and treatment through Project ECHO, developed public awareness campaigns (initiated in 2019), and initiated harm reduction programs, including medication assisted treatment (MAT) clinics (initiated in 2019) and syringe service programs (initiated in 2022) (17–20). In 2020, 5 years after launching the HCV elimination program, 71% of CNHS patients who were linked to HCV care initiated treatment and 80% of those who initiated treatment returned for a sustained virologic response at 12 weeks (SVR-12) visit (21). Additionally, 99% of those who returned for an SVR12 visit were cured (22). Despite these successes, the program has gaps in reaching its care and treatment goals.

Increasing linkage to HCV testing and treatment among underserved people who experience disparities in access, including those with SUD or inject drugs, is imperative to reach HCV elimination in Cherokee Nation. Community-based HCV screenings, including screenings conducted in homeless shelters, correctional facilities, mobile medical clinics, and other facilities, may enhance the reach and care of people who are inadequately served by traditional clinics or hospitals (22–31). Community-based HIV and syphilis screenings have also been shown to increase screening rates in underserved populations who may experience stigma, such as men who have sex with men living with or without HIV, persons who inject drugs, and those with a prior history of syphilis (32–36). CNHS previously showed that testing at food distribution sites improved access to screening and identification of community members with unmet healthcare needs, including HCV screening (31). Community-based screenings for those with SUD and/or with reduced access to healthcare is an important component of eliminating HCV in the Cherokee Nation.

The overall objective of this project was to describe the first 9 months of implementation of a community-based program to screen and link people with SUD and/or with access to healthcare issues to HCV, HIV, and syphilis care and treatment. Additional objectives were to describe the characteristics of people accessing the program, including their demographics, previous HCV, HIV, and syphilis screening and diagnoses, and risk factors; and identify characteristics associated with receiving confirmatory HCV, HIV, and syphilis testing.

## 2 Materials and methods

### 2.1 Setting

Cherokee Nation is the largest federally recognized tribe in the U.S. with more than 450,000 citizens. The reservation spans all or portions of 14 counties in northeastern Oklahoma (37). While a third of Cherokee citizens reside within the reservation, a large portion of the population in those counties are non-AI/AN individuals or are citizens of other tribes. CNHS is the largest tribally operated health system in the U.S., receiving over 1.6 million patient visits in 2023 (37). To be eligible for health services at no personal cost through CNHS, individuals typically must show proof of AI/AN descent, such as a Certificate of Degree of American Indian or Alaska Native Blood (CDIB) card or tribal enrollment or citizenship card from a federally recognized tribe (38). While most healthcare services at CNHS are restricted to AI/AN people, as this was a federally funded project, CNHS could provide screenings to both AI/AN and non-AI/AN people.

Cherokee Nation’s HCV Engagement and Linkage Program (CN HELP) initiative is a collaboration between CNHS and a harm reduction program, local men’s shelter, peer recovery support program, and a MAT program to provide HCV, HIV, and syphilis screenings. All collaborating programs are located in Tahlequah, Oklahoma (the capital of Cherokee Nation) except the MAT program. The MAT program is located in a nearby town within the Cherokee Nation reservation. These programs serve approximately 1,400 individuals annually, over 75% of whom are of AI/AN descent. CNHS staff members held once-a-week screenings at the harm reduction and peer recovery sites and as requested at the homeless shelter, depending on client turnover. On days when CNHS was not onsite, organization staff members referred individuals to the Cherokee Nation Infectious Disease Department (CN IDD) for screening. Thus, individuals who presented at the CN IDD clinic on their own record were eligible for screening on days the CN IDD clinical staff were not present at the community organizations. The MAT program screened and treated patients utilizing onsite healthcare staff, with technical support from CN IDD staff.

### 2.2 HCV, HIV, and syphilis screenings

#### 2.2.1 Eligibility

All people 18 years and older who visited the collaborating organizations from January through September 29, 2024 and who were able and willing to provide consent were eligible to participate in the screenings.

#### 2.2.2 Recruitment and enrollment

To recruit participants, staff members at participating organizations approached clients to inform them about the screening opportunity. If the client was receptive, the staff member brought them to a private room to receive information on eligibility, services offered, evaluation activities, and to complete consent procedures. During the consent process, eligible individuals received the option to screen for HCV and/or HIV/syphilis. The point-of-care (POC) tests utilized for this project were the Oraquick HCV Antibody and Chembio HIV/Syphilis Combo tests. All POC tests were collected via fingerstick, allowing multiple tests to be completed from a single sample, and could provide results within 20 min. All reactive POC tests required a confirmation test via venous blood draw before treatment. All confirmation testing occurred at the CN IDD, as program staff did not have the capacity to collect biosamples in the field. This required all participants with reactive POC test results to schedule an appointment at the CN IDD for further follow-up and evaluation. All samples were sent to a reference laboratory for confirmation testing and thus were unable to provide same day results. All participants who received a confirmation test was evaluated by a provider during the follow-up appointment.

In May 2024, to increase uptake of confirmation testing and linkage to care, if needed, the CNHS implemented a contingency management intervention. All enrolled participants were offered $10 gift cards at various points for their participation: completion of screening tests, confirmation laboratory testing for participants with reactive results, and treatment initiation for participants with active infections. In total, participants could receive up to $30 in gift cards.

AI/AN individuals with a reactive POC test were eligible for confirmation testing and subsequent treatment initiation at the CN IDD, located in Tahlequah, Oklahoma, between 0.4 and two miles away from each participating site.

Non-AI/AN individuals with a reactive HCV POC test were referred to an outside health system in Tulsa, Oklahoma for further evaluation, located 71 miles away from the CN IDD, and to the Cherokee County Health Department for further HIV or syphilis evaluation, located 4.6 miles away from the CN IDD.

CNHS clinical staff members recorded all screening results in the CNHS electronic medical record system. Reactive screening tests among AI/AN individuals triggered a follow-up appointment at the CN IDD for confirmatory testing and evaluation if the participant was receptive to scheduling.

### 2.3 Data collection

#### 2.3.1 Demographics

Program staff extracted demographic information for all patients from the CNHS electronic medical records including age in years, sex, and AI/AN status. AI/AN status was based on the individual’s eligibility to receive services at CNHS, as described above.

#### 2.3.2 Clinical measures

Program staff also extracted clinical information for AI/AN participants related to HCV, HIV, and syphilis from the CNHS electronic medical records including: POC test results, confirmation testing completion status, confirmation test results, and treatment initiation. Active infections were defined as follows and were based on confirmation test results: HCV, a quantifiable amount of HCV RNA ≥ 15 IU/mL; HIV, a positive confirmatory HIV-1/HIV-2 antibody differentiation immunoassay; Syphilis, a positive *Treponema pallidum* (TP) antibody plus a reactive Rapid Plasma Reagin (RPR) or two different TP antibody reactive test results without a prior history of adequate syphilis treatment. Regardless of previous history of HCV or syphilis infections, all participants with a positive antibody test were referred to confirmatory testing to rule out reinfection or relapse. Treatment initiation was based on whether medication was ordered and picked up or administered to the individual. For non-AI/AN individuals, staff members reviewed an internal CN HELP database to assess confirmation of laboratory testing, and treatment information, as this information was self-reported with documentation by the participants.

#### 2.3.3 Survey measures

All participants were eligible to complete a paper-based evaluation survey for an additional $20 gift card, which included questions about demographic characteristics (education, income, and marital status); previous testing for HCV, HIV, and syphilis; previous diagnosis of HCV, HIV, and syphilis infection; and risk factors for infection, including housing status, injection drug use (IDU) and sexual history (Tables 1, 2).

**Table 1:** Participant demographics, prior screening, and risk factors for HCV, HIV, and syphilis, 2024.

Characteristic, No. (%) ^a^	Participants (*N* = 503)
Age, years	
18–34	161 (32)
35–54	259 (52)
55+	81 (16)
Sex	
Female	239 (50)
Male	236 (49)
Other	5 (1)
AI/AN	
Yes	365 (73)
No	138 (27)
Education	
Less than high school	128 (26)
High school or GED	186 (39)
More than high school	165 (35)
Income	
≤$15,000	319 (67)
>$15,000	158 (33)
Marital status	
Married or living with partner	230 (48)
Divorced/separated/widowed	83 (17)
Single	163 (34)
Ever tested for HCV	
Yes	226 (46)
No	202 (41)
Do not know/not sure	59 (14)
Ever tested for HIV	
Yes	288 (58)
No	174 (35)
Do not know/not sure	36 (7)
Ever tested for Syphilis	
Yes	204 (41)
No	235 (47)
Do not know/not sure	60 (12)
Ever had HCV	
Yes	54 (11)
No	429 (86)
Do not know/not sure	15 (3)
Ever had HIV ^b^	
Yes	NA
No	NA
Do not know/not sure	NA
Ever had Syphilis	
Yes	42 (8)
No	442 (88)
Do not know/not sure	19 (4)
Living in transitional housing, staying in a shelter, or experiencing homelessness	
Yes	153 (30)
No	350 (70)
Do not know/not sure	0 (0)
Lifetime IDU	
Yes	220 (45)
No	259 (53)
Do not know/not sure	10 (2)
Recent IDU	
≤6 Months ago	118 (25)
>6 Months ago	108 (23)
Never injected drugs	196 (41)
Do not know/not sure	56 (12)
Shared needles	
Yes	82 (17)
No	202 (42)
Never used needles	168 (35)
Do not know/not sure	26 (5)
Condom use at most recent sexual encounter	
Yes	75 (15)
No	364 (74)
Do not know/not sure	50 (10)

HCV, Hepatitis C; AI/AN, American Indian and Alaska Native; GED, General Education Development program; IDU, Injection Drug Use.

a Percentages may not add to 100% due to rounding.

b HIV counts and percentages are withheld due to small sample sizes with potential for identifiability.

**Table 2:** Demographics and risk factors of participants who completed HCV and syphilis confirmation testing, 2024.

Characteristic, No. (%)^a^	HCV Confirmation Completed (*n* = 102)		*p* value	Syphilis Confirmation Completed (*n* = 60)		*p* value
	Yes	No		Yes	No	
Age, years						
18–34	8 (40)	12 (60)	0.158	7 (26)	20 (74)	0.533
35–54	15 (27)	40 (73)		10 (40)	15 (60)	
55+	13 (48)	14 (52)		2 (40)	3 (60)	
Sex						
Female	15 (41)	22 (60)	0.923	11 (32)	23 (68)	0.894
Male	20 (36)	35 (64)		7 (37)	12 (63)	
Other	1 (33)	2 (67)		1 (50)	1 (50)	
AI/AN						
Yes	35 (51)	34 (49)	<0.0001	17 (37)	29 (63)	0.304
No	1 (3)	32 (97)		2 (18)	9 (82)	
Education						
Less than High School	14 (48)	15 (52)	0.261	3 (23)	10 (77)	0.252
High school or GED	11 (41)	16 (59)		4 (21)	15 (79)	
More than high school	10 (29)	25 (71)		10 (45)	12 (55)	
Income						
≤$15,000	29 (36)	51 (64)	0.969	18 (41)	26 (59)	0.075
>$15,000	5 (36)	9 (64)		1 (9)	10 (91)	
Marital status						
Married or living with partner	11 (31)	25 (69)	0.403	7 (24)	22 (76)	0.210
Divorced/separated/widowed	8 (50)	8 (50)		2 (25)	6 (75)	
Single	16 (38)	26 (62)		9 (50)	9 (50)	
Living in transitional housing, staying in a shelter, or experiencing homelessness						
Yes	15 (44)	19 (56)	0.201	8 (36)	14 (64)	0.268
No	15 (28)	38 (72)		7 (25)	21 (75)	
Do not know/not sure	4 (50)	4 (50)		2 (67)	1 (33)	
Lifetime IDU						
Yes	26 (36)	46 (64)	0.630	9 (33)	18 (67)	0.611
No	7 (32)	15 (68)		7 (27)	19 (73)	
Do not know/not sure	0 (0)	3 (100)		-	-	
Recent IDU						
≤6 Months ago	13 (33)	27(68)	0.808	6 (35)	11 (65)	>0.999
>6 Months ago	13 (42)	18 (58)		4 (31)	9 (69)	
Never injected drugs	2 (29)	5 (71)		7 (35)	13 (65)	
Do not know/not sure	5 (29)	12 (71)		1 (25)	3 (75)	
Shared needles						
Yes	16 (50)	16 (50)	0.258	7 (50)	7 (50)	0.454
No	16 (32)	34 (68)		6 (29)	15 (71)	
Never used needles	1 (17)	5 (83)		5 (28)	13 (72)	
Do not know/not sure	1 (20)	4 (80)		0 (0)	1 (100)	
Condom use at most recent sexual encounter						
Yes	10 (50)	10 (50)	0.251	6 (35)	11 (65)	>0.999
No	21 (33)	43 (67)		11 (31)	24 (69)	
Do not know/not sure	4 (25)	12 (75)		2 (40)	3 (60)	

HCV, Hepatitis C; AI/AN, American Indian and Alaska Native; GED, General Education Development program.

a Percentages may not add to 100% due to rounding.

#### 2.4 Data analysis

Data were analyzed using descriptive statistics including counts, proportions, and graphs. To compare demographic characteristics associated with completing confirmation testing, group comparisons were performed by Chi-square test or Fisher’s exact test, as indicated. Analyses were performed using SAS 9.4 (39).

## 3 Results

### 3.1 Participant demographics, previous screenings, and risk factors

Overall, there were 503 participants (Table 1). Among them, 259 (52%) were 35 to 54 years of age, 239 (50%) were female, and 365 (73%) were AI/AN. Almost two-fifths of participants reported having a high school diploma or GED, approximately two-thirds indicated they had an income of $15,000 or less, and nearly half reported being married or living with a partner.

Among all participants, 226 (46%) reported previous HCV testing, 288 (58%) reported previous HIV testing, and 204 (41%) reported previous syphilis testing (Table 1). There were 54 (11%) participants who reported ever having HCV and 42 (8%) participants who reported ever having syphilis. Participants who reported ever testing positive for HIV were suppressed from reporting due to a small sample size. However, there were no individuals newly diagnosed with HIV.

Among the assessed risk factors, 153 (30%) participants reported that they currently live in transitional housing, are staying in a shelter, or are experiencing homelessness (Table 1). In addition, 220 (45%) participants reported lifetime IDU, with 118 (25%) reporting IDU within the previous 6 months. Further, 82 (17%) participants reported using a needle after someone else had used it to inject drugs. Finally, 364 (74%) participants reported not using condoms during their most recent sexual encounter, with an additional 50 (10%) reporting that they did not know/were unsure.

### 3.2 HCV screening testing, confirmation testing, and treatment initiation results

**Figure 1:**
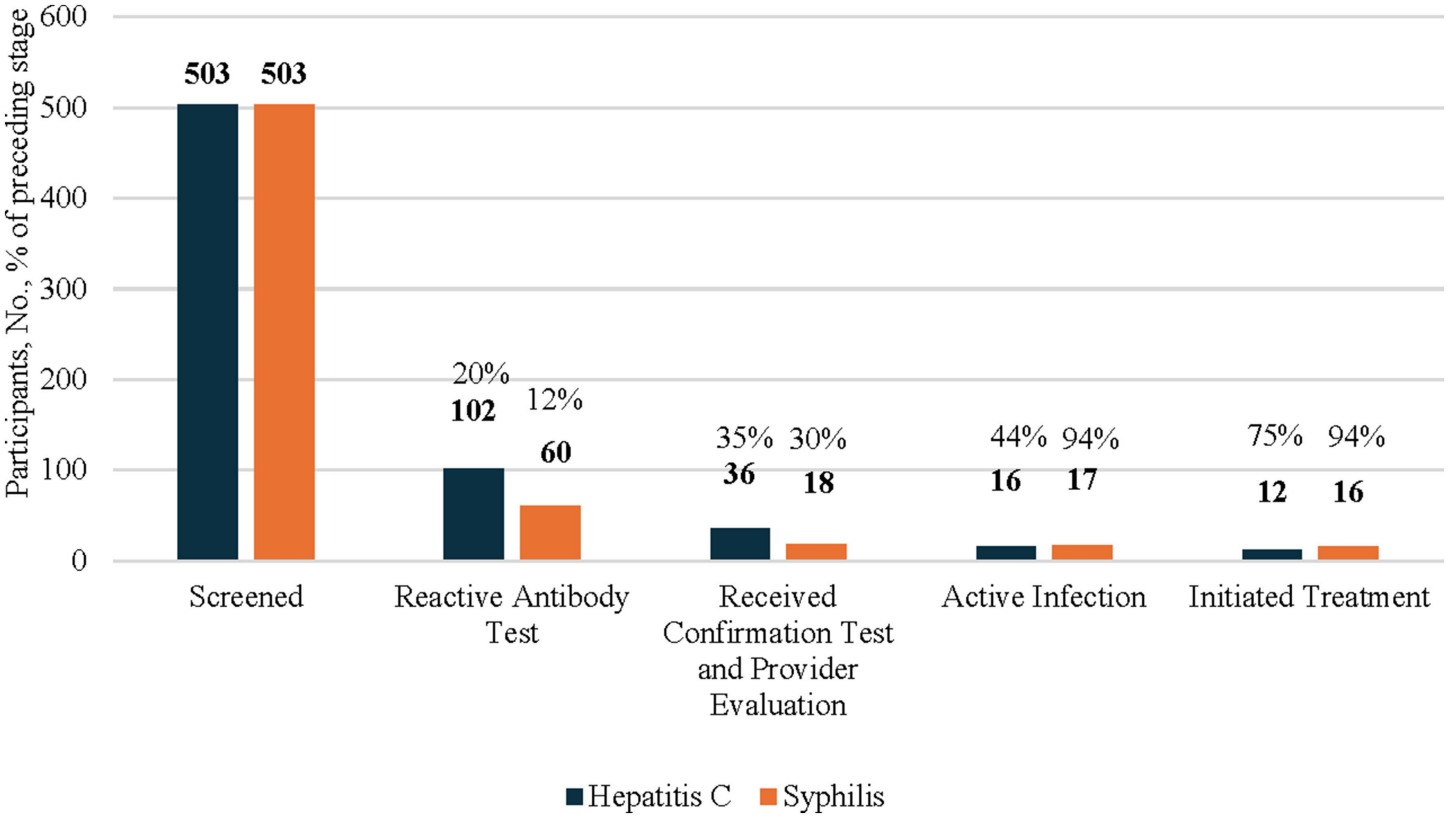
HCV and syphillis screening testing, confirmation testing, and treatment initiation results among all participants 2024. This figure’s data include all participants of the CN HELP Initiative through the first 9 months of the program. Percentages of each columns are a proportions of the prior column.

**Figure 2:**
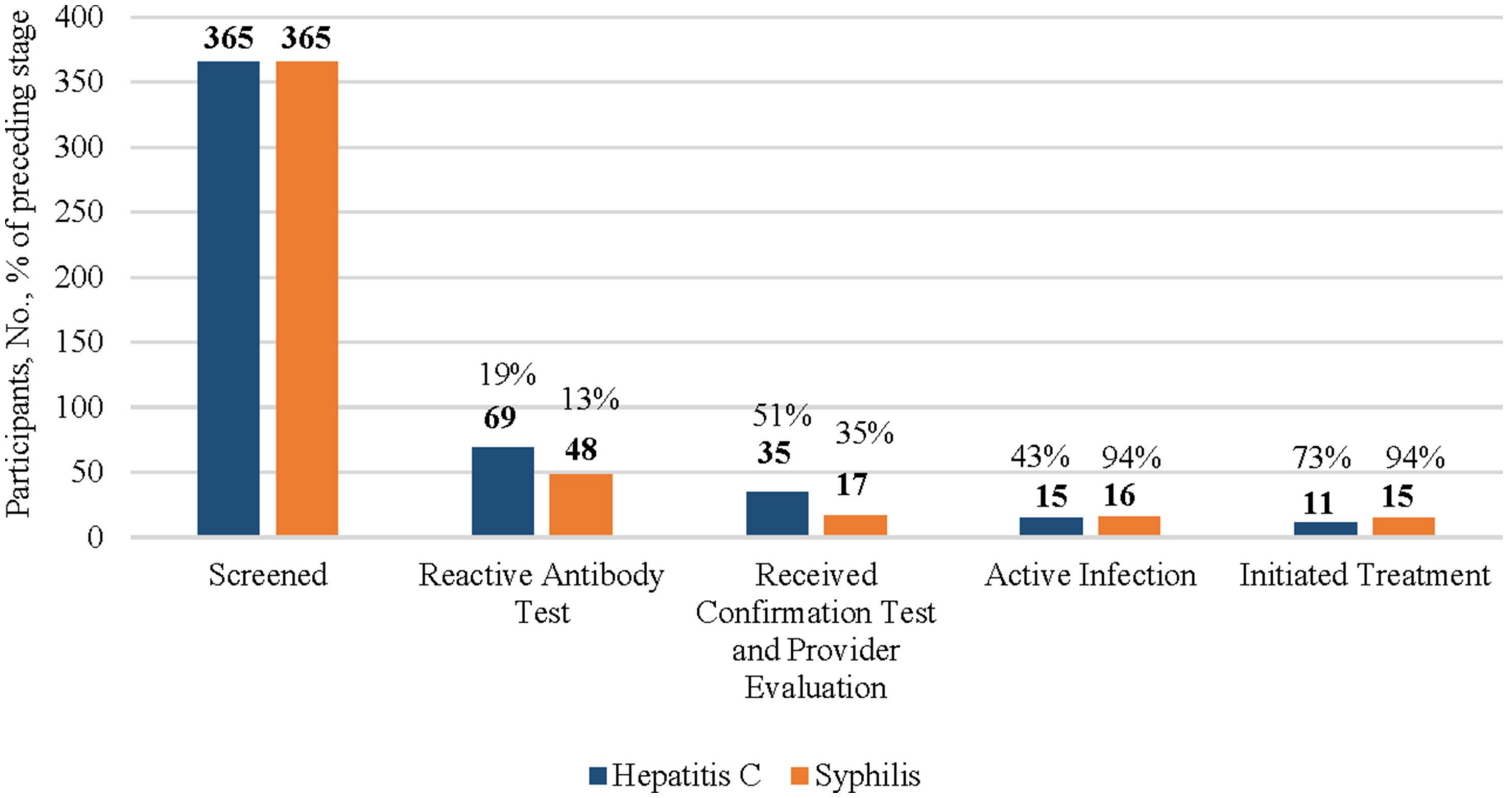
HCV and syphillis screening testing, confirmation testing, and treatment initiation results among American Indian and Alaska Native (AI/AN) participants, 2024. This figure’s data include all AI/AN participants of the CN HELP Initiative through the first 9 months of the program. Percentages of each columns are a proportions of the prior column.

Program staff screened 503 people (Figure 1), including 365 AI/AN people (Figure 2), for HCV. Among those screened, 102 (20%) had a reactive HCV antibody test (Figure 1). Of those with a reactive HCV antibody test, 36 (35%) underwent HCV RNA confirmation testing, with 16 (44%) diagnosed with an active infection, among whom 12 (75%) initiated treatment. As a note, 24 of the 66 individuals who did not receive confirmation testing during the study period, received confirmation testing at later date after CNHS implemented the HCV POC RNA testing option. Of those 24, 13 (54%) tested positive for HCV and 5 (38%) initiated treatment (data not included in figures, data not shown).

Among the 365 AI/AN participants who were screened for HCV, 69 (19%) had a reactive HCV antibody test (Figure 2). Of those with a reactive HCV antibody test, 35 (51%) were linked to care and received HCV RNA confirmation testing, with 15 (43%) diagnosed with an active infection, among whom, 11 (73%) initiated treatment.

### 3.3 HIV screening testing, confirmation testing, and treatment initiation results

Among the 503 people (Figure 1), including 365 AI/AN people (Figure 2) who were screened for HIV, less than 1% of individuals had a reactive HIV antibody test. Due to a small sample size, the number of people with HIV and who were linked to HIV care were suppressed. All individuals with a reactive test were already aware of their HIV-positive status and most reported viral suppression. These individuals were tested for HIV because they wished to receive syphilis testing, and the test utilized was an HIV/syphilis combination test.

### 3.4 Syphilis screening testing, confirmation testing, and treatment initiation results

Among the 503 people who were screened for syphilis, 60 (12%) had a reactive TP antibody test (Figure 1). Of these, 18 (30%) received confirmation testing, 17 (94%) of whom were found to have active untreated syphilis, and 16 (94%) initiated treatment.

Among the 365 AI/AN syphilis screening participants, 48 (13%) had a reactive TP test, among whom 17 (35%) underwent confirmation testing (Figure 2). Of the 16 AI/AN individuals diagnosed with an active untreated infection, 15 (94%) initiated treatment.

### 3.5 Demographic and risk factor characteristics of participants by HCV and syphilis confirmation testing completion

A statistically significant higher percentage of AI/AN people (51%) received HCV confirmation testing compared with non-AI/AN people (3%) (Table 2). There were no other statistically significant associations between demographic and risk factor characteristics and receipt of HCV or syphilis confirmation testing. Due to a small sample size, stratification was not performed for HIV confirmation testing.

## 4 Discussion

Findings from this project highlight the potential impact of community-based screening programs in reaching AI/AN individuals and people with reduced access to healthcare, including people who inject drugs. The CNHS community screening program primarily reached individuals aged 35–54 years with low income, and served a balanced mix of sexes, education levels, and marital statuses. While many had previously been tested for HIV, fewer had undergone prior testing for HCV or syphilis. Importantly, most participants diagnosed with active HCV or syphilis infection initiated treatment, underscoring the value of immediate access to confirmation testing and an evaluation by an HCV provider. Although no new HIV cases were identified, the program’s ability to identify individuals in need of care for other infections with similar risk factors supports the inclusion of this screening in future intervention strategies. Finally, most people who were screened through the initiative were AI/AN, suggesting that similar community-based efforts can support engagement of this population.

Notably, AI/AN identity was the only demographic factor significantly linked to successful HCV confirmatory testing completion. The higher completion of confirmation testing observed among AI/AN participants (HCV: AI/AN: 51%, Non-AI/AN: 3%; syphilis: AI/AN: 37%, Non-AI/AN: 18%) may reflect the program’s successful tribally led outreach, but it may also reflect the presence of a nearby tribal health infrastructure supporting continuity of care. The presence of a nearby health system underscores the importance of the availability of accessible healthcare for this population. However, the relatively low rates of prior HCV, HIV, and syphilis testing (46, 58, and 41%, respectively) among this population, even for those with relatively close healthcare access, highlight the ongoing gaps in screening within this population.

Our findings align with the literature, demonstrating the success of community-based screenings for HCV, HIV and syphilis on reaching people with SUD and/or with reduced access to healthcare (22–36). Project findings indicate that the CN HELP Initiative reached a population with limited access to, or engagement in, routine HCV, HIV, and syphilis testing and treatment. The high proportion of participants without stable housing, along with reported behaviors, such as lifetime IDU, needle sharing, and inconsistent or uncertain condom use, highlight the need to provide additional support for this population. These findings reinforce the critical need for community-based interventions that address both infectious disease screening and the broader context of harm reduction and prevention. Although the percentage of participants reporting lifetime IDU (45%) was lower than anticipated, participants visit these sites for a variety of resources and self-reported data on stigmatized and/or illegal activities are often underreported. Despite this, the percentage of participants who reported lifetime IDU was much higher than a previous CNHS community-based screening assessment (8%) conducted across 340 people accessing two food distribution sites in Tahlequah and Sallisaw, Oklahoma in 2019 (31).

Though this program was successful at reaching individuals with a history of SUD for screening, low uptake of confirmation testing and further evaluation was observed as a challenge, despite the short distance to the CN IDD for AI/AN individuals. It is not surprising that a higher percentage of AI/AN compared with non-AI/AN participants received confirmation testing for HCV (AI/AN: 51%; Non-AI/AN: 3%) and syphilis (AI/AN: 37%; Non-AI/AN: 18%). While AI/AN people receive healthcare at no personal cost and access to a clinic nearby through CNHS, non-AI/AN participants are referred to the local county health department, several miles away, and another agency located more than 70 miles away, for services. Strengthening referral services and connecting all community members to accessible healthcare should be a priority for community-based screening programs. While AI/AN individuals are priority for tribes and tribal organizations, elimination goals may not be reached without serving all non-AI/AN community members given that these infections are contagious.

The findings of this project are subject to several limitations. First, small sample sizes of reactive tests may have limited power to detect differences in confirmatory testing by subgroup, especially among non-AI/AN participants. As the CN HELP Initiative continues and the number of screenings increases, future analyses should be conducted to reassess demographic differences in confirmation testing and treatment initiation. Second, Cherokee Nation is not a closed population and there is the possibility that participants received healthcare outside of CNHS, leading to incomplete follow-up information. Third, this project relied on self-reported information, which is subject to recall bias and social desirability bias, which may have led to underreporting of risk factors across all participants and confirmation testing and treatment initiation information for non-AI/AN participants. Finally, the study relies on sex instead of gender, and did not collect information on sexual orientation, both of which have been associated with risk of acquiring these infections (40).

This project also has several strengths including adding to the literature demonstrating that community-based screening programs are successful for AI/AN, along with non-AI/AN, communities. This project also included a substantial sample size of AI/AN people, which is not common across the literature and is necessary given the unique circumstances of many AI/AN communities in terms of culture, history, land, legal systems, access to resources, and access to healthcare, among many other factors. Finally, through strong partnerships between CNHS and the community-based organizations and MAT program, this project demonstrated the importance of collaboration in meeting the needs of community members who may have challenges accessing traditional healthcare services.

Since initiating the Cherokee Nation HELP program, the U. S. Food and Drug Administration has approved the first HCV RNA POC test, which CNHS has since implemented. Additionally, in year two of the CN HELP program, CNHS has been able to build capacity to conduct onsite blood draws for confirmation testing with the hire of a phlebotomist. Further, CNHS now has onsite clinical staff (nurse and advance practice provider) for further patient evaluation as indicated and is working to obtain approvals for onsite treatment options. Although the impact of these changes on increasing confirmation testing and treatment remain to be seen, preliminary analyses suggest that onsite confirmation testing options have increased the number of individuals who know their infection status. Future studies should assess the impact of incorporating HCV RNA POC testing in tribal community-based settings, as well as how tribally led programs may improve participation in screening and treatment programs. In addition, future studies assessing factors that reduce or improve engagement in care and treatment among people in tribal settings with SUD and/or experiencing other factors that may hinder access to healthcare should be prioritized, as it may be helpful for developing specific interventions to improve patient outcomes.

## 5 Conclusion

Overall, the CN HELP Initiative was successful in screening individuals for HCV, HIV, and syphilis with moderate success in linking them to follow-up evaluation and treatment initiation, especially for AI/AN individuals. Future interventions should include onsite confirmation test and treatment options to prevent barriers to accessing healthcare.

## Data Availability

The datasets presented in this article are not readily available because as a Sovreign Nation, Cherokee Nation reserves the right to restrict access to tribal data. Access to data may be given if all appropriate tribal approvals are obtained. Anyone interested in data access should contact the corresponding author. Requests to access the datasets should be directed to AC, ashley-comiford@cherokee.org.
